# Enteropathogenic *E. coli* effectors EspF and Map independently disrupt tight junctions through distinct mechanisms involving transcriptional and post-transcriptional regulation

**DOI:** 10.1038/s41598-018-22017-1

**Published:** 2018-02-27

**Authors:** Anand Prakash Singh, Swati Sharma, Kirti Pagarware, Rafay Anwar Siraji, Imran Ansari, Anupam Mandal, Pangertoshi Walling, Saima Aijaz

**Affiliations:** Centre for Molecular Medicine, Jawaharlal Nehru University, New Delhi, 110067 India

## Abstract

Enteropathogenic *E. coli* infection is characterized by rapid onset of diarrhea but the underlying mechanisms are not well defined. EPEC targets the tight junctions which selectively regulate the permeability of charged and uncharged molecules. Cooperative actions of the EPEC effectors EspF and Map have been reported to mediate tight junction disruption. To analyze the individual contributions of EspF and Map, we generated *in vitro* models where EspF and Map, derived from the EPEC strain E2348/69, were constitutively expressed in epithelial cells. Here we report that tight junction disruption by EspF and Map is caused by the inhibition of the junctional recruitment of proteins during tight junction assembly. Constitutive expression of EspF and Map depleted the levels of tight junction proteins. EspF down-regulated the transcript levels of *claudin-1, occludin* and *ZO-1*, while Map down-regulated only *claudin-1* transcripts. Both effectors also caused lysosomal degradation of existing tight junction proteins. We also identified a novel interaction of Map with non-muscle myosin II. Consistent with earlier studies, EspF was found to interact with ZO-1 while actin was the common interacting partner for both effectors. Our data provides evidence for the distinct roles of Map and EspF in tight junction disruption through non-synergistic functions.

## Introduction

Enteropathogenic *E. coli* (EPEC) is a leading cause of infant diarrhea in developing countries^1,2^. EPEC colonizes the intestinal epithelial cells and uses a type III secretion system to translocate more than twenty effector proteins into the host cells^3^. EPEC infection is characterized by the increased permeability of solutes through the intestinal epithelial cells. The cells of the intestinal epithelium adhere to each other through adhesive complexes which include tight junctions (TJ), adherens junctions and desmosomes^4,5^. TJs are the most apical of the junctional complexes and are crucial for the formation of a semi-permeable barrier that selectively regulates the passage of charged and uncharged molecules^4,5^. TJs are complex structures that consist of transmembrane proteins as well as a cytoplasmic plaque comprising of proteins that are involved in multiple cellular functions including the regulation of permeability, polarity, cell adhesion, proliferation and differentiation^4,5^. The transmembrane proteins of the TJs include claudins, occludin, tricellulin and junctional adhesion molecules which contain extracellular domains involved in the sealing of adjacent cells^4–6^. The C-termini of the transmembrane proteins are linked to cytoplasmic plaque proteins which include the zonula occludens (ZO) proteins, kinases, phosphatases, GTPases, exchange factors and transcriptional and post-transcriptional regulators^4–6^. These cytoplasmic plaque proteins are in turn linked to the actin cytoskeleton and serve as a connection between the transmembrane proteins and the peri-junctional actinomyosin ring. Permeability through TJs is primarily regulated by claudins and occludin although cytoplasmic plaque proteins such as the ZO proteins and exchange factors that activate Rho GTPases have also been linked to the regulation of permeability^4–7^. While occludin and claudins directly regulate the permeability of uncharged and charged molecules respectively, the adaptor protein ZO-1 regulates this process through the modulation of the actin cytoskeleton^4–8^.

TJ disruption is a common feature associated with microbial pathogenesis^9,10^. EPEC also targets the TJ complex leading to the displacement of several TJ proteins and increased permeability through the intestinal epithelium^2,3^. Of the many effectors translocated into the host cell by EPEC, only EspF, EspG1/G2, Map and NleA have been so far reported to disrupt the TJ barrier^11–13^. However, little is known about the molecular mechanisms employed by these effectors to disrupt the TJs. One limitation has been the non-availability of a suitable model system that mimics the process of human infection. So far, studies to understand the molecular basis of EPEC-mediated TJ disruption have relied either on *in vivo* models (infections of rabbits and mice with the related rabbit (REPEC) or mouse (*C. rodentium*) strains of EPEC) or *in vitro* models (infections of cultured epithelial cells with the human EPEC strain E2348/69)^14^. These studies have provided significant insights into the pathogenesis of EPEC infections. For example, i*n vivo* mouse models where *C. rodentium* was used to infect mice revealed the process of attaching/effacing pathogenesis in greater detail^15^. Studies conducted in other *in vivo* mouse models, where EPEC infected C57BL/6 J mice were used, revealed that these mice were susceptible to EPEC infection and later studies showed that EPEC-mediated TJ disruption was accompanied by the displacement of occludin and ZO-1 from the membrane to the cytoplasm while a mutant EPEC strain lacking EspF had no effect on the barrier function indicating an important role of EspF in mediating TJ disruption^16–18^. *In vitro* models using HeLa, Caco-2 or T84 cells infected with wild type EPEC have revealed that EPEC infection decreases transepithelial resistance, a measure of TJ integrity, and increases electrolyte transport^19–21^. Using these *in vitro* models, EPEC was shown to dislocate occludin from the TJs which was mediated by EspF^22,23^. Subsequent studies using polarized Caco-2 cells revealed that the EPEC effector Map can also disrupt TJs independently of EspF^11^. This was further confirmed by infection of HeLa cells with *C. rodentium* as well as in mice infected with *C. rodentium*^24^. However, conflicting data exists regarding the roles of EspF and Map in barrier disruption. Some studies have indicated that synergistic actions of Map and EspF are required for TJ dysfunction; other studies have suggested that Map might exert a greater effect on TJs and another *in vivo* study using *C. rodentium* has shown that EspF but not Map plays an important role in the disruption of the TJ barrier *in vivo*^11,25,26^. Therefore, the independent roles of EspF and Map in disrupting TJs are unclear.

EspF is a 206 amino acid protein that contains an N-terminal mitochondrial targeting signal (amino acids 1–24), a nucleolus targeting signal (amino acids 21–74) and three proline-rich repeats (PRR) at the C-terminus^27^. Map is a 203 amino acid protein which is also targeted to the mitochondria and has been reported to induce transient filopodia^28,29^. Several lines of evidence suggest that EspF and Map may not have redundant functions. For example, EspF has been reported to disrupt the host cell mitochondria and nucleolus, remodel the plasma membrane by binding with sorting nexin 9 (SNX9) and promote actin polymerization by binding and activation of neural Wiskott-Aldrich syndrome protein (N-WASP)^27,30^. Its role in causing diarrhea has been linked to its ability to disrupt TJs, inactivate the sodium hydrogen exchanger 3 (NHE3) and in association with the bacterial surface protein Intimin and its receptor Tir inactivate the sucrose glucose cotransporter-1 (SGLT-1)^11,25,27,31^. Map also disrupts the host mitochondria, inactivates SGLT-1 and increases TJ permeability^11,25^. Additionally, Map interacts with Na^+^/H^+^ exchanger regulatory factors 1 and 2 (NHERF1, -2)^32,33^. These studies indicate that except for their effect on mitochondrial and SGLT-1 functions, EspF and Map do not target the same signaling pathways. We therefore hypothesized that EspF and Map might also have distinct roles in junctional barrier defects.

In order to examine the individual contributions of EspF and Map on TJ integrity, we generated an *in vitro* model in which the gene encoding EspF or Map, derived from EPEC O127:H6 strain E2348/69, was fused N-terminally with EGFP and stably integrated into MDCK (Madin-Darby Canine Kidney) cells for the constitutive expression of these effectors. We used the MDCK cell line as it is a prototypic cell line for the study of TJ assembly. MDCK cells are widely used to study the biology of influenza virus^34–36^. MDCK cells have also been employed for understanding the cellular basis of EPEC infection. For example, MDCK and HEK (human embryonic kidney) cells were successfully infected with wild type EPEC to show the role of host cell plasma membrane phosphoinositides on EPEC adherence^37^. Similarly, MDCK cells infected with wild type EPEC have been used to show the recruitment of the podosome-specific scavenger protein Tks5 to the site of EPEC attachment in a Tir-dependent manner^38^. Additionally, earlier studies have reported the use of MDCK cells to examine the effect of EPEC infection on TJ barrier resistance and permeability^20^. Elegant studies involving transient and regulated expression of EPEC effectors in MDCK cells have revealed the involvement of EspG in TJ disruption^12,39^ and the role of EspF on cell polarity and membrane trafficking^30,40^. We recently reported that constitutive expression of Map in an *in vitro* MDCK model is sufficient to disrupt TJs^41^. We now report the generation of a similar model for EspF and have used both the EspF and Map *in vitro* models to examine the roles of these effectors on TJ barrier function. The idea behind this study was to delineate the individual functions of these effectors on epithelial TJs in a bacteria-free system with no interference from other EPEC effectors. Here, we demonstrate that EspF and Map are independently sufficient to disrupt TJ integrity. Both effectors prevent the recruitment of occludin and claudin(s) into TJs during junction assembly leading to the aberrant localization of the proteins in the cytoplasm and their eventual depletion. A comparison of relative gene expression profiles of *claudin-1*, *claudin-4* and *occludin* in the stable cell lines expressing EspF and Map indicated that these effectors have distinct roles in transcriptional and post-transcriptional regulation of TJ proteins. Co-immunoprecipitation experiments confirmed the previously reported interaction of EspF with ZO-1 and that of both effectors with actin^42,43^ highlighting that the MDCK model is comparable to other *in vitro* models. Further, we identified a novel interaction of Map with non-muscle myosin II. Our data suggests that EspF and Map target functionally distinct signaling pathways to mediate TJ disruption.

## Results

### Constitutive expression of EspF and Map disrupts TJ integrity in MDCK cell lines

**Figure 1:**
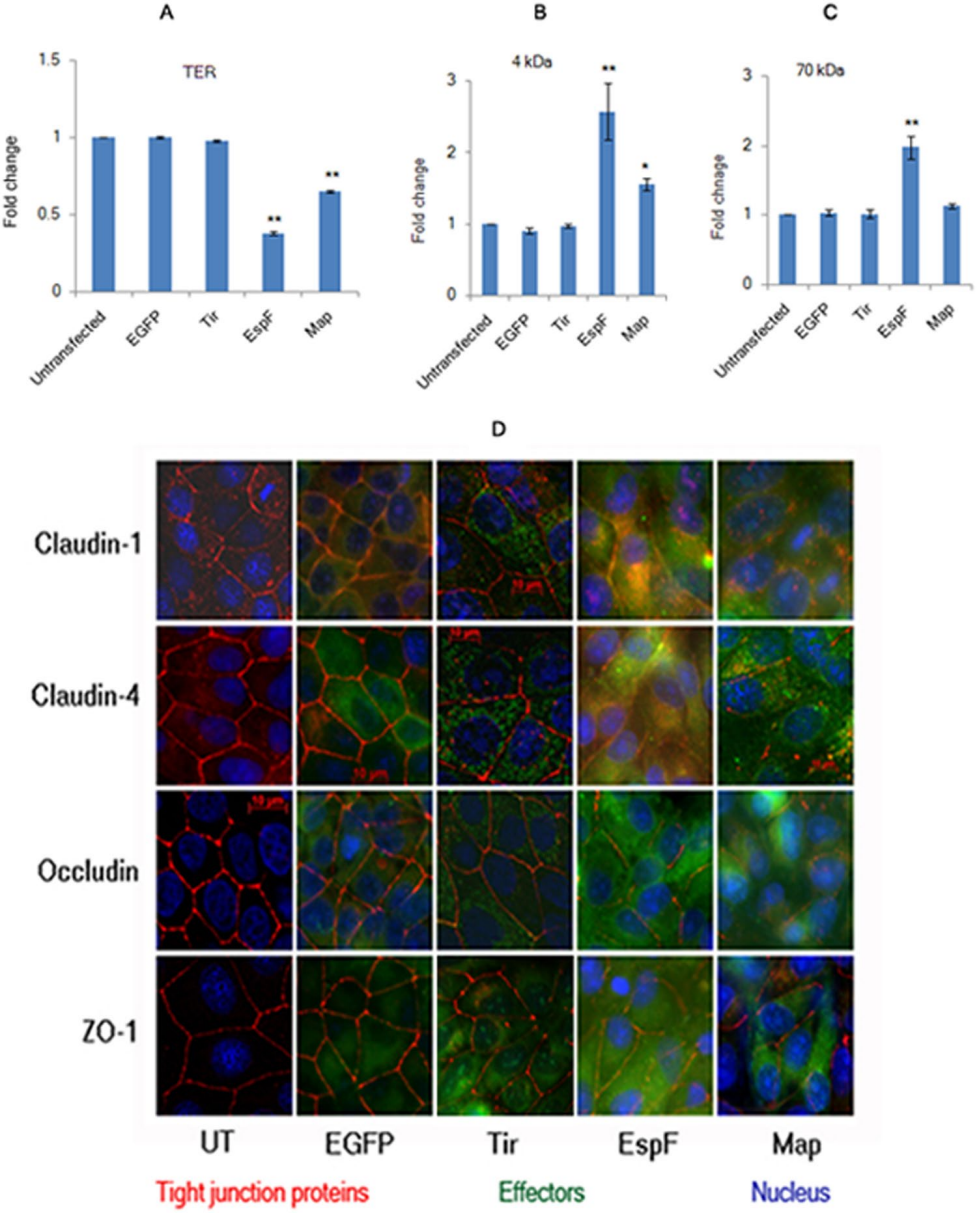
Constitutive expression of EGFP-EspF and EGFP-Map in MDCK cells disrupts the tight junction barrier. (**A**) Untransfected cells or cells constitutively expressing EGFP vector alone, EGFP-Tir, EGFP-EspF and EGFP-Map were cultured on polycarbonate Transwell filters and TER was measured daily to monitor the integrity of tight junctions. The maximum values of TER for each cell line are indicated. The paracellular flux of 4 kDa dextran (**B**) and 70 kDa dextran (**C**) was measured and plotted as fold change with respect to untransfected MDCK cells (normalized to 1). Error bars represent means ± s.e.m from three independent experiments; *indicates p-value < 0.01 and **indicates p-value < 0.001. (**D**) The cellular localization of TJ proteins was examined using the indicated primary antibodies and Cy3-conjugated secondary antibodies. Nucleus was labeled with DAPI (4′,6-Diamidino-2-phenylindole dihydrochloride). Constitutive expression of EGFP-EspF and EGFP-Map caused the reorganization of claudin-1, -4, occludin and ZO-1. Scale: 10 µm; UT: Untransfected.

The genes encoding EspF and Map, derived from the human EPEC strain E2348/69, were cloned in pEGFP-C1 vector to generate N-terminal EGFP-tagged EspF and Map constructs. These constructs were then stably integrated into MDCK cells to generate stable cell lines constitutively expressing EspF or Map. We first confirmed the validity of EspF and Map *in vitro* MDCK models by assessing the effect of these proteins on transepithelial resistance (TER) and paracellular permeability in confluent cells. Stable cell lines expressing EGFP-EspF or EGFP-Map were grown on permeable Transwell filters until fully confluent monolayers were formed. Untransfected MDCK cells (UT), stable cells lines expressing empty pEGFP-C1 vector (EGFP) and stable cells lines expressing EGFP-tagged Tir (Translocated Intimin Receptor, Tir) were used as controls. TER was measured daily for all samples and maximum values were attained in 7 days for all cell lines (Fig. 1). The maximum TER values of EspF and Map cell lines were determined and compared with the maximum TER values obtained for untransfected MDCK cells. Cells expressing EGFP-EspF and EGFP-Map displayed maximum TER values which were only 0.38 fold and 0.65 fold of the maximum TER values of untransfected cells respectively (Fig. 1A). Cells expressing either EGFP vector alone or EGFP-Tir displayed TER values comparable to untransfected cells. We next evaluated the effect of EspF and Map on the flux of small and large uncharged tracers (4 kDa and 70 kDa dextran). As compared to untransfected cells (normalized to 1), cells expressing EspF exhibited an increase of 2.57 fold in the flux of the 4 kDa tracer and 1.97 fold in the flux of the 70 kDa tracer (Fig. 1B,C). Map expression increased the flux of 4 kDa dextran by 1.55 fold and had no significant effect on the flux of 70 kDa dextran (Fig. 1B,C). The permeability of 4 kDa and 70 kDa dextran in cells expressing EGFP vector and EGFP-Tir was not significantly different from untransfected cells. These data indicate that, consistent with previous studies, both effectors increased the permeability of charged and uncharged molecules. Since the TJ constituent proteins claudin(s), occludin and ZO-1 have been linked to the regulation of TJ permeability, we examined the cellular localisation of these proteins in cells constitutively expressing EGFP-EspF and EGFP-Map. Claudins belong to a large family comprising of at least 24 members which are often co-expressed. While some members of the claudin family play a role in adhesion, others regulate the permeability of ions^44^. In this study, we have focussed on claudin-1 and claudin-4 since these claudins have been reported to play an important role in the regulation of TJ barrier function in MDCK cells^45,46^. Cellular localisation using specific antibodies revealed that claudin-1 and −4 were localised at mature TJs in all the control cell lines (untransfected, EGFP and Tir) but not in cells expressing EspF and Map where these claudins were found to be predominantly accumulated in the cytoplasm with reduced staining at TJs. The staining of occludin was observed to be markedly discontinuous and reduced at TJs in EspF and Map cell lines while ZO-1 staining appeared to be marginally reduced but was normally localised at TJs (Fig. 1D). These observations are consistent with studies in other *in vitro* models which have shown that EspF and Map reorganise TJ proteins^22,23,42^. To examine if EspF and Map had the same effect upon ectopic expression in intestinal epithelial cells, we expressed EspF and Map in the intestinal epithelial cell line, T84. Fully confluent monolayers of T84 cells (with well assembled TJs) were transiently transfected with plasmid constructs encoding EGFP-EspF or EGFP-Map and the cellular localisation of TJ proteins was examined after 24 hours. Similar to MDCK cells, we found that claudin-1, -4 and occludin showed reduced staining at TJs while ZO-1 staining was only marginally affected (Supplementary Figure S1). Control cells expressing EGFP vector or EGFP-Tir displayed normal junctional localization of TJ proteins in T84 cells.

### EspF and Map inhibit *de novo* tight junction assembly

**Figure 2:**
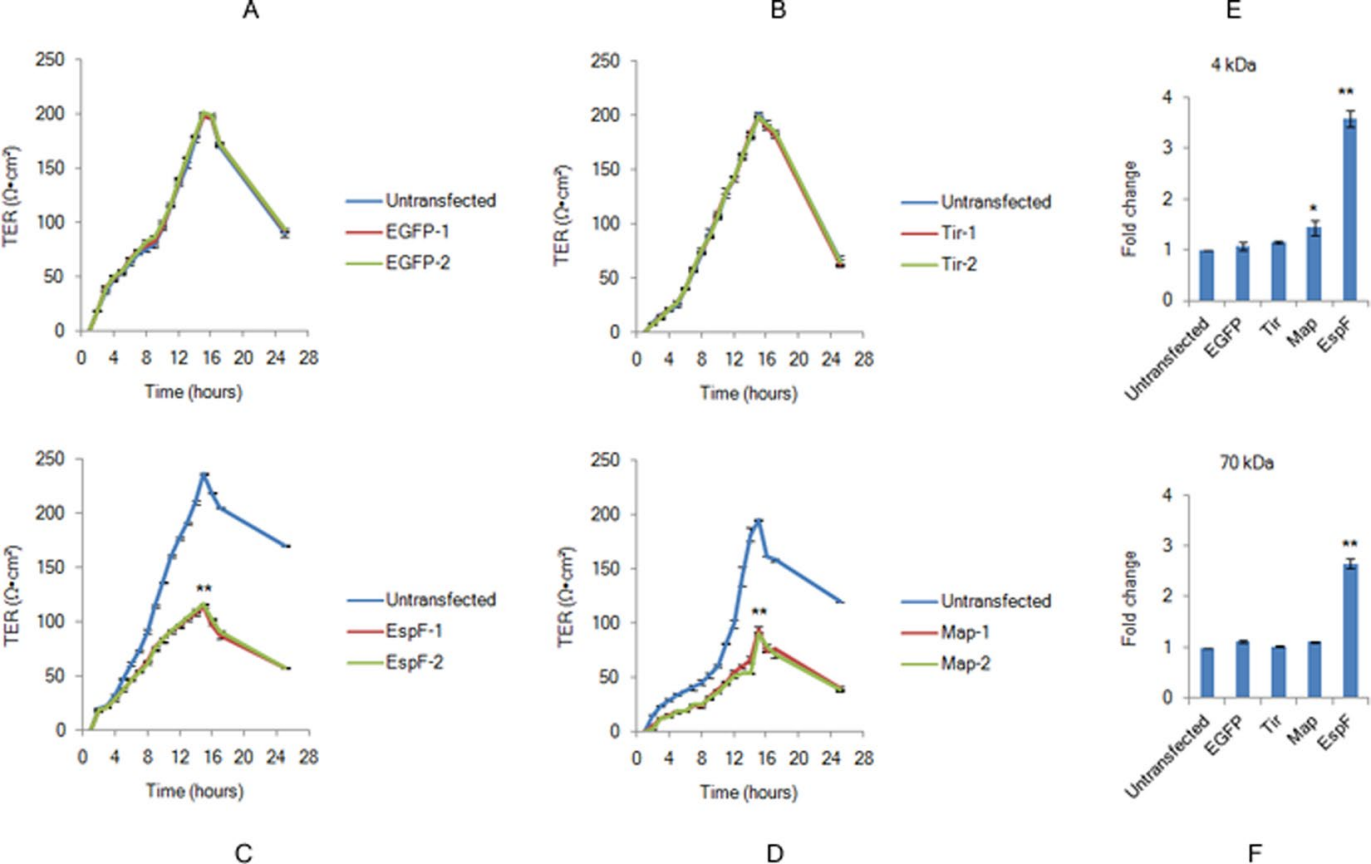
EspF and Map inhibit *de novo* tight junction assembly. (**A**–**D**) Untransfected, EGFP, EGFP-Tir, EGFP-EspF or EGFP-Map cells were grown on permeable filters in low calcium (<5 µM) medium for 18–20 hours and then switched to normal calcium (~1.8 mM) medium to initiate junction assembly. TER was measured hourly for 24 hours. EGFP-1, EGFP-2; Tir-1, Tir-2; EspF-1, EspF-2, Map-1 and Map-2 represent biological replicates. Data are represented as means ± s.e.m. from three independent experiments with 4 filters (n = 4) taken for each cell line; **indicates p-value < 0.001. (**E**,**F**) The paracellular flux of 4 kDa and 70 kDa dextran tracers was measured in untransfected, EGFP, EGFP-Tir, EGFP-EspF and EGFP-Map cell lines. Shown are fold changes calculated with respect to untransfected cells (normalized to 1). Error bars represent means ± s.e.m from three independent experiments; *represents p-value < 0.01 and **represents p-value < 0.001.

The assembly of TJs is a dynamic process involving continuous remodeling events where existing junctional proteins are internalized and replaced with new proteins^47^. In order to examine if TJ biogenesis is affected in the presence of EspF and Map, we employed the calcium switch model^48^. This model has been reported to mimic the process of epithelial morphogenesis and is widely used to study the *de novo* assembly of TJs in MDCK cells in a synchronised manner^49^. In this method, cells are first maintained in low calcium (<5 µM) medium to disrupt cell-cell junctions and apical-basolateral polarization and then switched to normal calcium (1.8 mM) medium in which the cells rapidly polarize and develop intercellular junctions^49^. Earlier studies on this model have revealed that in MDCK cells, maximum TER values are attained within 12–16 hours of the addition of normal calcium medium due to the exocytotic fusion of the TJ components at the plasma membrane, after which the TER drops to steady state values of 50–60 Ωxcm^2^ in these cells^48,50^. To determine whether EspF and Map can independently interfere with junction dynamics, all cell lines were grown on membrane filters in low calcium medium for 18–20 hours to disrupt existing junctions. Junction assembly was then initiated by the addition of normal calcium medium. The assembly of TJs was monitored by measuring TER after each hour. Cells constitutively expressing EGFP alone or EGFP-Tir showed TER values comparable to untransfected MDCK cells indicating that neither the EGFP vector nor a control EPEC effector (Tir) affected junction assembly (Fig. 2A,B). The TER values in the two biological replicates of EspF-expressing cell lines were ~48% and ~49% (Fig. 2C) while those of Map-expressing cell lines were ~46% and ~48% (Fig. 2D) as compared to untransfected cell lines. The starting TER values in the first 2 hours after switching to normal calcium medium were 20 ± 4 Ωxcm^2^ for all cell lines. However, after 2 hours the TER values in EspF and Map cells lines lagged behind the TER values of untransfected cells. We next determined the extent to which the junctional pore size had increased due to the expression of EspF or Map. After the completion of the TER experiment, the cells lines were incubated with fluorescently labeled dextran tracers of 4 kDa and 70 kDa. As compared to untransfected cells (normalized to 1), EspF cell lines exhibited an increase of ~3.6 fold and ~2.7 fold in the permeability of the 4 kDa dextran and 70 kDa dextran respectively (Fig. 2E,F). In contrast, Map cell lines exhibited an increase of ~1.4 fold in the permeability of the 4 kDa tracer and had no effect on the permeability of the 70 kDa tracer (Fig. 2E,F). Taken together, these data indicate that both effectors decrease TER to a similar extent during junction assembly but their role in increasing the permeability of non-charged tracers is markedly different with EspF exerting a more significant effect than Map. These effects were specific to Map and EspF as EGFP-tagged Tir did not have any significant effect on the permeability of 4 kDa and 70 kDa tracers (Fig. 2E,F).

### EspF and Map prevent junctional recruitment of proteins during *de novo* TJ assembly

**Figure 3:**
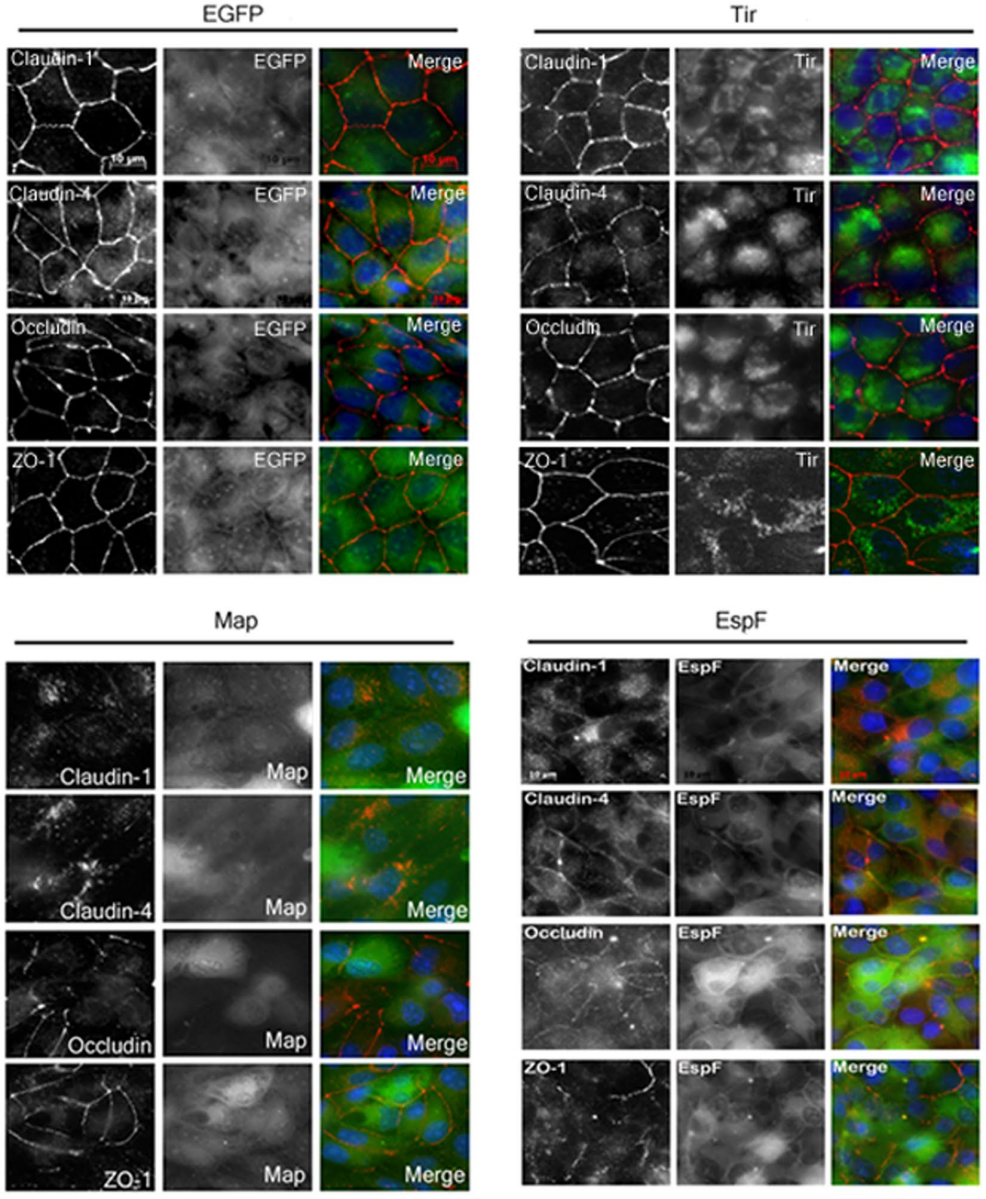
EspF and Map prevent the junctional recruitment of proteins during *de novo* junction assembly. EspF and Map, but not Tir, prevent the incorporation of proteins in forming junctions. Cells were cultured on cover-slips in low calcium (<5 µM) medium for 18–20 hours and then switched to normal calcium (~1.8 mM) medium. After 14 hours, cells on cover-slips were fixed and stained with primary antibodies against the indicated tight junction proteins and Cy3-conjugated secondary antibodies. Nucleus was labeled with DAPI (4′,6-Diamidino-2-phenylindole dihydrochloride). Cover slips were mounted on glass slides and images were acquired on a Zeiss ApoTome (Axiovert 40 CFL) using 63X oil objective. (n = 3); scale: 10 µm.

We next assessed the cellular localization of TJ proteins during *de novo* junction assembly in EGFP, Tir, EspF and Map cell lines. After 14 hours in normal calcium medium (when maximum TER values were obtained for all cell lines), junctional recruitment of proteins was found to be complete in untransfected, EGFP and Tir cell lines but not in EspF and Map cell lines which showed an aberrant distribution of TJ proteins. The expression of EspF and Map itself was localized in the cytoplasm as well as the plasma membrane of cells (Fig. 3). In EspF cell lines, claudin-1 and claudin-4 were mostly localized in the cytoplasm. For occludin and ZO-1, only discontinuous localization was observed at the TJs (Fig. 3). In the Map cell lines, claudin-1 and −4 were localized in the cytoplasm in punctate structures while occludin and ZO-1 were recruited to the tight junctions but their expression was low and junctional localization was discontinuous (Fig. 3). Cells expressing either EGFP vector alone or EGFP-Tir showed normal localization pattern of all the TJ proteins. These data indicate that EspF and Map compromise junction integrity by inhibiting the recruitment of TJ proteins into newly forming junctions.

### Constitutive expression of EspF and Map depletes the levels of tight junction proteins

**Figure 4:**
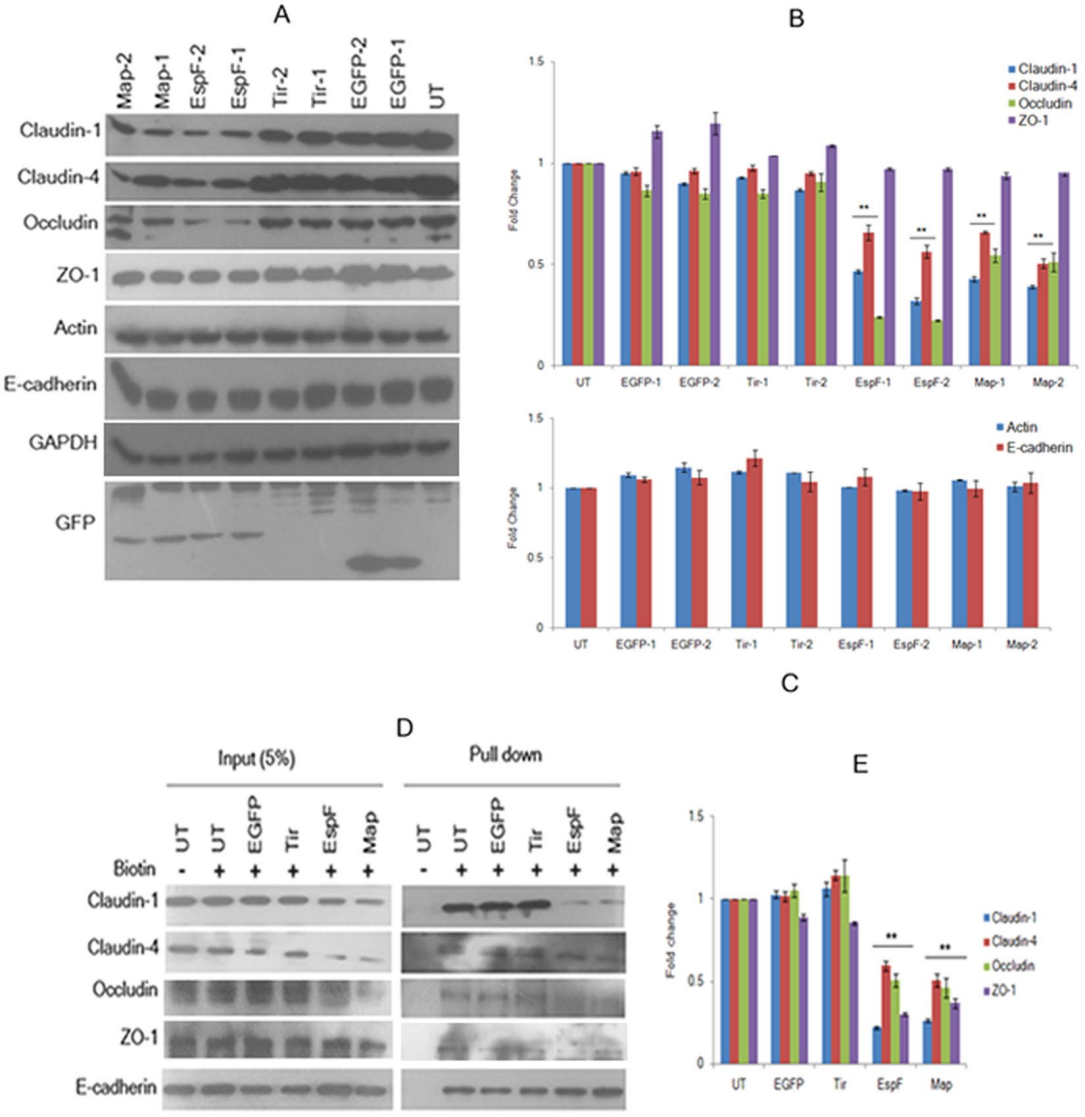
Constitutive expression of EspF and Map depletes tight junction proteins. (**A**) Cell lysates derived from untransfected, EGFP, EGFP-Tir, EGFP-EspF and EGFP-Map cell lines were separated by electrophoresis on 12% SDS-polyacrylamide gels and transferred to PVDF membranes. Equal amounts of cell lysates were loaded after normalizing with GAPDH. Blots were probed with the indicated primary antibodies and HRP-conjugated secondary antibodies. Full-length blots are presented in Supplementary Figure S3. (**B**,**C**) The x-ray films were scanned and quantitative analysis was performed by measuring band intensities using ImageJ software. The expression of different tight junction proteins in these cell lines was normalized with respect to untransfected cells and fold changes were calculated. Data are represented as means ± s.e.m. from six independent experiments using two biological replicates for all cell lines; **represents p-values < 0.001. (**D**) The cell surface proteins were labeled with membrane impermeable Sulfo-NHS-Biotin and immobilized on streptavidin-agarose beads. The beads were washed and subjected to SDS-PAGE. E-cadherin was used as a loading control for cell surface proteins. Full-length blots are presented in Supplementary Figure S4. (**E**) The levels of cell surface proteins were estimated by densitometric scanning of x-ray films using ImageJ software. Shown are fold changes with respect to untransfected cells. Error bars represent means ± s.e.m. from three independent experiments; **represents p-values < 0.001; UT: Untransfected.

As the immunofluorescence data suggested that EspF and Map expression caused reduced staining of proteins at the TJs, we hypothesized that EspF and Map might be either inhibiting the expression of TJ proteins or preventing their integration into the TJ complex without affecting total levels. We first assessed the total levels of junctional proteins in cell lysates derived from untransfected, EGFP, Tir, EspF and Map cell lines (Fig. 4). The total levels of TJ proteins showed a significant decrease in EspF and Map cell lines but not in cell lines expressing EGFP alone or EGFP-Tir (Fig. 4A,B). In the two Map cell lines, the levels of claudin-1, claudin-4, and occludin were reduced to ~0.4 ± 0.04 fold, ~0.6 ± 0.16 fold, and ~0.5 ± 0.03 fold respectively as compared to untransfected cells while no significant reduction was observed for ZO-1 (Fig. 4A,B). Similarly, in the two EspF cell lines, the levels of claudin-1, claudin-4, and occludin were reduced to ~0.4 ± 0.15 fold, 0.6 ± 0.1 fold, and 0.2 ± 0.16 fold respectively as compared to untransfected cells (normalized to 1) while no significant reduction was seen for ZO-1 (Fig. 4A,B). This depletion was specific for TJ proteins as EspF or Map expression had no effect on the levels of the adherens junction protein E-cadherin or on β-actin which had expression levels comparable to untransfected cells (Fig. 4A,C). In order to determine if this depletion occurred at the TJ complex, we labeled the cell surface proteins with membrane impermeable Sulfo-NHS-Biotin and isolated the biotin-tagged proteins using immobilized streptavidin-agarose resin. The samples were subjected to SDS-PAGE and E-cadherin was used as a cell surface loading control as E-cadherin levels are not affected by either EspF or Map. As shown in Fig. 4, there was a significant reduction in the levels of biotin-labeled TJ proteins in cells expressing EspF and Map. The levels of biotin-labeled claudin-1, claudin-4, occludin and ZO-1 were found to be reduced to ~0.2, ~0.6, ~0.5 and ~0.3 fold in EspF expressing cells and ~0.3, ~0.5, ~0.5 and ~0.4 fold in Map expressing cells respectively (Fig. 4D,E). In cells expressing either EGFP or EGFP-Tir the levels of biotin-labeled TJ proteins were similar to untransfected cells (Fig. 4D,E). These data indicate that EspF and Map not only deplete the total levels of claudin-1, -4 and occludin but also inhibit their junctional recruitment leading to their depletion at the plasma membrane. Biotin labeling also revealed that although there was no decrease observed in the total levels of ZO-1 in EspF and Map cell lines (Fig. 4A,B), biotin-labeled ZO-1 levels were reduced at the plasma membrane.

### EspF and Map regulate the expression of tight junction proteins through transcriptional and post-transcriptional mechanisms

**Figure 5:**
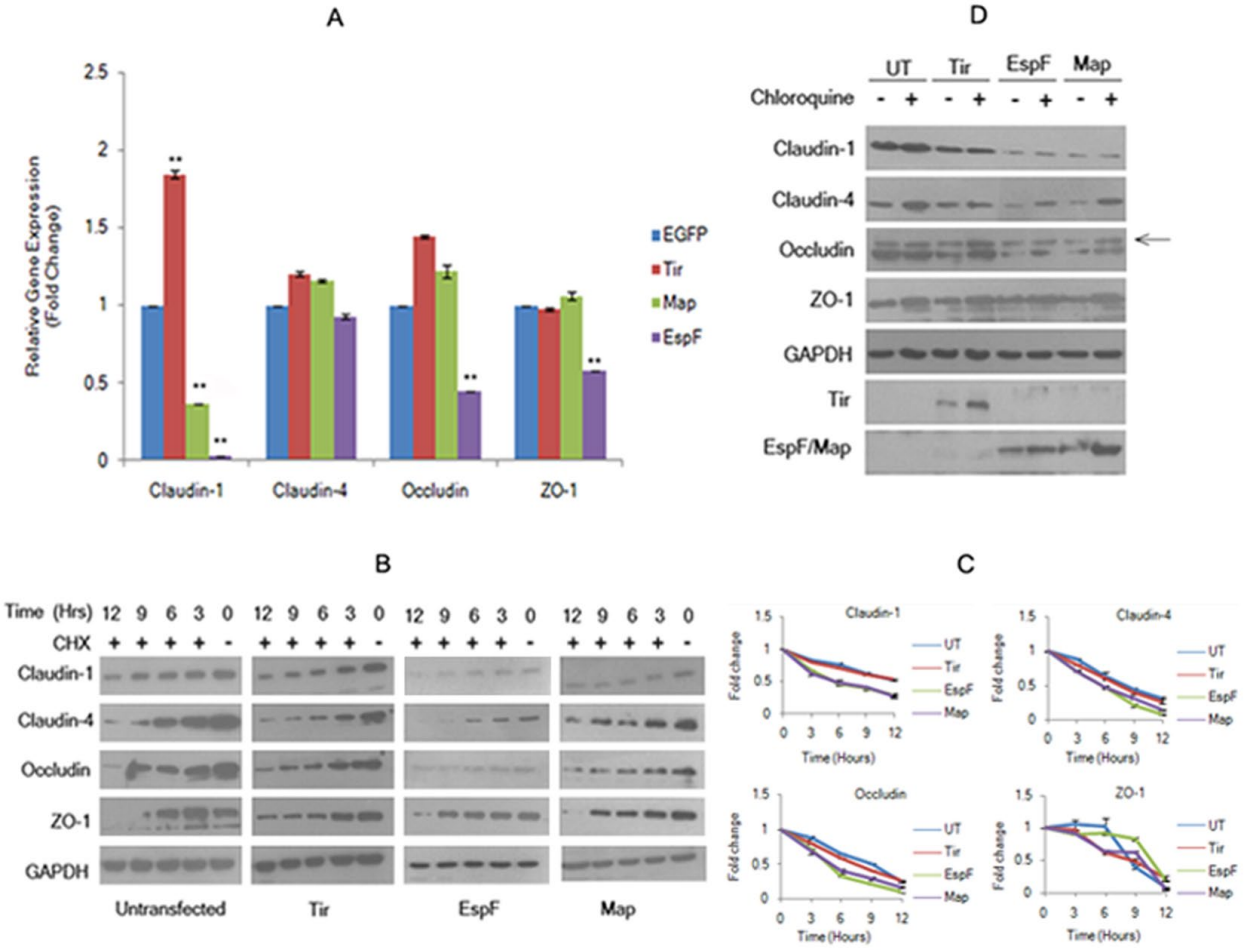
Map and EspF modulate the transcriptional and post-transcriptional regulation of tight junction proteins. (**A**) Relative quantitation of gene expression. Relative expression of *claudin-1*, *claudin-4*, *occludin* and *ZO-1* in cells expressing EGFP, Tir, EspF and Map was determined by qPCR using TaqMan gene expression assays. Shown are fold changes. Data are represented as means ± s.e.m. from three independent experiments; **represents p-value < 0.001. (**B**,**C**). Expression of EspF and Map affects the stability of TJ proteins. Confluent cultures of cells were treated with cycloheximide (25 μg/ml) for 3, 6, 9 and 12 hours prior to cell lysis and analyzed by immuno-blotting with the indicated primary antibodies. Full-length blots are presented in Supplementary Figure S5. (**C**) The fold changes with respect to untransfected cells (normalized to 1) were calculated by estimating the band intensities using ImageJ software and plotted. (**D**) Effect of the lysosomal inhibitor chloroquine on the amounts of TJ proteins. Confluent cultures of cells were treated with 100 µM chloroquine for 18 hours and cell lysates were analyzed by immuno-blotting with the indicated antibodies. Experiments were performed three times and representative blots are shown. UT: Untransfected. Full-length blots are presented in Supplementary Figure S6.

We next examined if the depletion in TJ proteins was regulated at the transcription or translation level. To determine if the depletion in protein levels was due to a down-regulation in the expression of the genes encoding these proteins, we performed quantitative real time PCR (qPCR) assays using primer/probes for *claudin-1*, *claudin-4*, *occludin* and *ZO-1*. The fold changes in the expression levels of the genes encoding these TJ proteins in EspF and Map cells, relative to those in cells constitutively expressing EGFP vector alone, were calculated by the ΔΔC_T_ method^51^. This method determines the relative gene expression in the target and reference samples by measuring fluorescence levels at a threshold cycle (C_T_). We used *GAPDH* as an endogenous control in qPCR reactions. In Map cell lines, we observed a decrease in the expression of *claudin-1* to ~0.36 fold, while no significant change was observed in the gene expression profiles of *claudin-4*, *occludin* and *ZO-1* as compared to EGFP cells (Fig. 5A). In EspF cell lines, the relative fold changes in the gene expression levels of *claudin-1*, *claudin-4*, *occludin* and *ZO-1* were found to be ~0.03, ~0.92, ~0.45, and ~0.58 fold respectively (Fig. 5A). These data indicated that Map significantly down-regulated the transcription of *claudin-1* but had no effect on the transcription of genes encoding other TJ proteins. On the other hand, EspF was found to inhibit the transcription of *claudin-1, occludin* and *ZO-1* significantly while the expression level of *claudin-4* was not decreased significantly. Our western blot data (Fig. 4) indicated that EspF did not deplete the levels of ZO-1 but the qPCR data showed a significant decrease in *ZO-1* transcripts. These differences may be a reflection of the different half-lives of the corresponding transcripts and proteins. Notably, *claudin-1* was the common target for both EspF and Map as both effectors decreased the transcription of *claudin-1*. We also observed a significant increase in the *claudin-1* transcripts in cells expressing Tir (Fig. 5A). It is unclear why Tir expression increases *claudin-1* transcripts. An earlier study, in which HA-tagged Tir was over-expressed in MDCK cells during infection, reported that over-expressed Tir tightens the junctions but did not identify the mechanism^37^. Our qPCR data suggests that one possible mechanism might be the up-regulation of *claudin-1* transcripts. As Tir is required for intimate attachment of EPEC to the host cells during infection, we speculate that this might be a mechanism to transiently stabilize the TJs until EPEC is firmly adhered to the host cell and all effectors have been translocated. However, confirmation of this hypothesis will require additional work.

As mentioned above, TJs are dynamic structures where TJ proteins undergo continuous endocytosis and are replaced with new proteins^47^. As the qPCR data suggested that transcriptional repression did not occur for *claudin-4*, *occludin* and *ZO-1* in Map expressing cells and to some extent on *claudin-4* in EspF expressing cells, we wanted to know why these proteins were depleted in these cells lines. To examine if EspF and Map expression interfered with the stability of the TJ proteins, all cell lines were treated with cycloheximide (25 µg/ml) to inhibit protein synthesis for 3, 6, 9 and 12 hours and total cell lysates were analyzed by SDS-PAGE and western blotting (Fig. 5B,C). The EGFP-Tir cell lines did not show significant differences in the stability of claudin-1, -4, occludin and ZO-1 from untransfected cells (Fig. 5B,C). However, the stability of these proteins was found to decline in EGFP-EspF and EGFP-Map expressing cells. About 50% depletion was observed in the amount of claudin-1, claudin-4 and occludin within 6 hours in EspF and Map cell lines as compared to 9 to 12 hours in untransfected or Tir cell lines while no significant difference was observed in the stability of ZO-1 (Fig. 5B,C). The TJ barrier is regulated by rapid endocytosis and recycling of the constituent proteins^52^. We therefore tested whether the lysosomal and/or proteasomal degradation pathways were being activated by EspF and Map. Addition of the proteasomal inhibitor MG132 did not increase the levels of the TJ proteins in either the Map or EspF expressing cell lines (data not shown). However, incubation of these cells lines with the lysosomal inhibitor, chloroquine, was found to increase the total levels of claudin-4 and occludin (Fig. 5D). The increase in the levels of ZO-1 was comparable to that seen in untransfected cells. Notably, no increase was observed in the levels of claudin-1 in both the EspF and Map cell lines. This is consistent with the qPCR data as claudin-1 is depleted at the transcription level in both cell lines. Although chloroquine treatment of EspF cell lines caused an increase in the total levels of claudin-4 and occludin, this increase was more pronounced in Map cell lines. This data further suggested that EspF-mediated depletion of TJ proteins occurred at the transcriptional and post-transcriptional levels while Map caused the depletion of the TJ proteins (except for claudin-1) by targeting them for lysosomal degradation (Fig. 5D).

### EspF and Map interact with distinct tight junction-associated proteins

**Figure 6:**
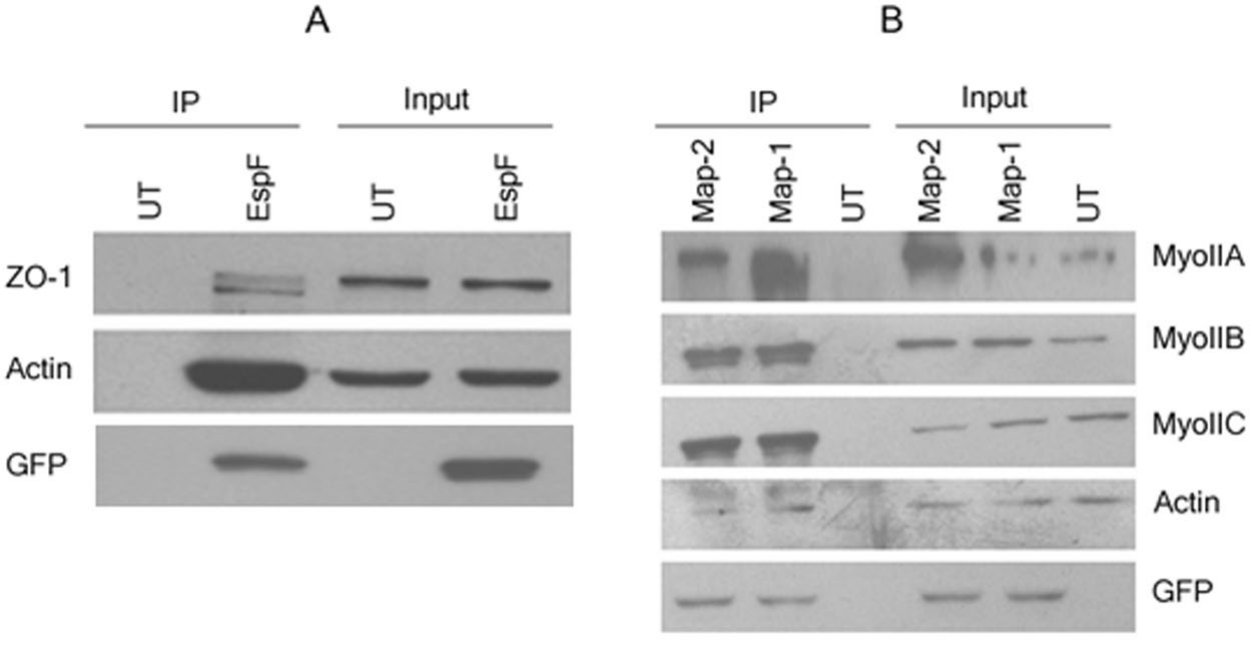
Map and EspF interact with distinct tight junction regulatory proteins. (**A**,**B**) Confluent cultures of untransfected, EGFP-EspF or EGFP-Map cells were lysed in 1 × RIPA buffer and the supernatant was incubated with rabbit anti-GFP antibody overnight at 4 °C. Interacting proteins were immuno-precipitated by mixing with 50 µl of protein G-agarose beads for 4 hours at 4 °C. After washing, the beads were subjected to western blot analyses (n = 3). Blots were probed with indicated primary antibodies. Experiments were performed three times and representative blots are shown. UT: Untransfected. Full-length blots are presented in Supplementary Figure S7.

We next examined whether EspF and Map interacted with TJ proteins to prevent their junctional recruitment. Co-immunoprecipitation and GST pull-down assays did not reveal any interaction of EspF and Map with occludin, claudin-1 or claudin-4, the major TJ proteins responsible for the regulation of permeability (data not shown). We then looked for interactions with other tight junction-based proteins that have been reported to indirectly regulate permeability. In co-immunoprecipitation assays performed using anti-GFP antibody, Map was found to interact with all the isoforms of non-muscle myosin II (Fig. 6B). Consistent with earlier published reports, EspF was found to interact with ZO-1 (Fig. 6A). No interaction with these proteins was observed in cell lysates derived from untransfected cells (Fig. 6A,B). Additionally, both EspF and Map were found to interact with actin (Fig. 6A,B). Actin plays a central role in regulating TJ permeability through interactions with ZO-1 and the motor protein non-muscle myosin II^8,53^. Thus it is likely that EspF disrupts TJs through ZO-1 mediated contractility of the actin cytoskeleton while Map exerts it effect through direct modulation of the actinomyosin complex.

### Tight junction disruption by EspF and Map is independent of mitochondrial function

**Figure 7:**
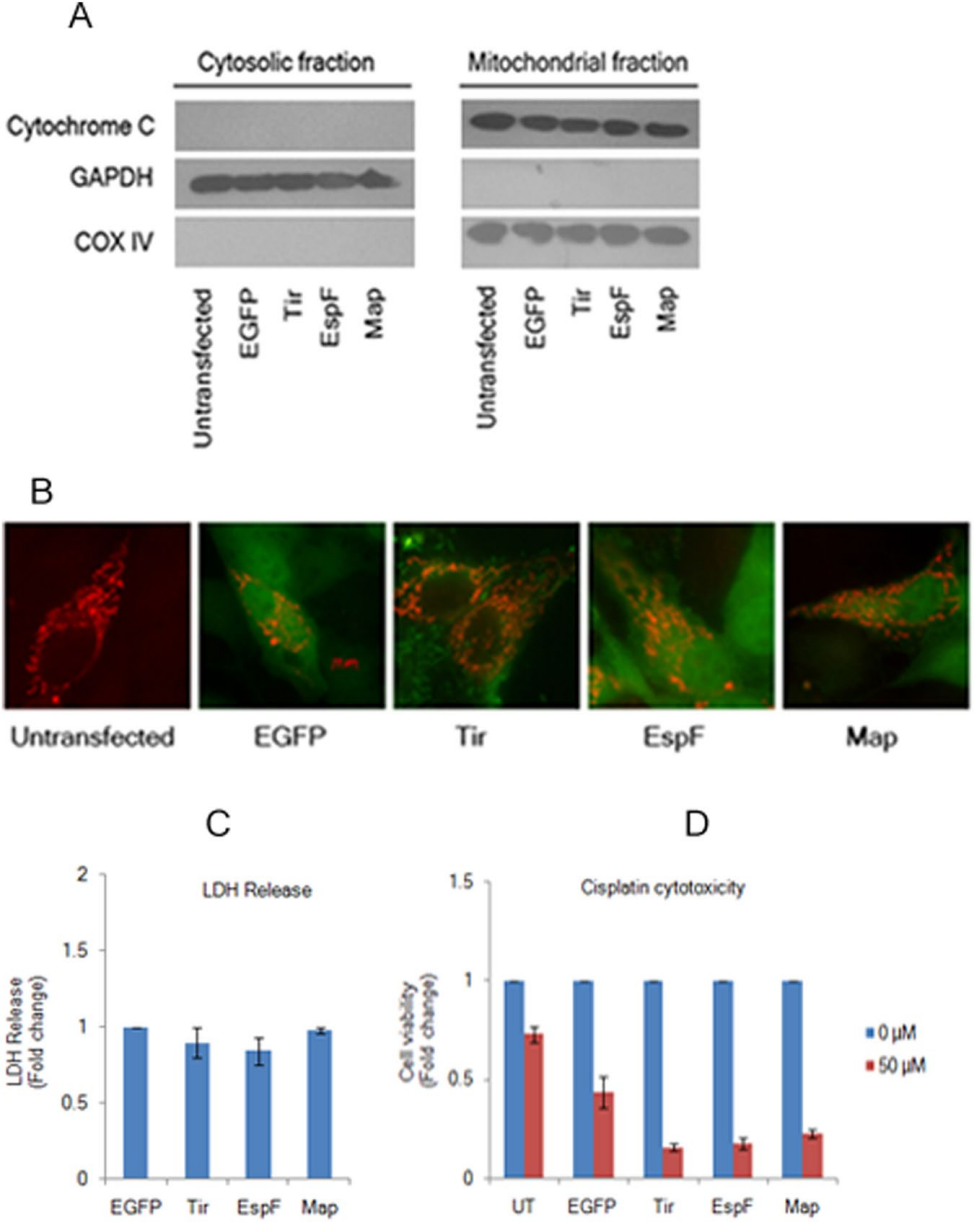
Tight junction disruption by EspF and Map is independent of mitochondrial function. (**A**) Constitutive expression of EGFP-EspF and EGFP-Map does not cause the release of cytochrome *c* from the mitochondria. Cytosolic and mitochondrial fractions were isolated from untransfected, EGFP, EGFP-Tir, EGFP-EspF and EGFP-Map cell lines and analyzed by immuno-blotting with cytochrome *c* antibody. Equal loading on gels was confirmed by using COX IV as the mitochondrial marker and GAPDH as the cytosolic marker. Blots are representative of three independent experiments. Full-length blots are presented in Supplementary Figure S8. (**B**) Cells expressing EGFP-EspF and EGFP-Map display normal mitochondrial morphology. Cells from untransfected, EGFP, EGFP-Tir, EGFP-EspF and EGFP-Map cell lines were grown on glass cover-slips and transduced with CellLight Mitochondria-RFP, BacMam 2.0 reagent to label the mitochondria with RFP. Cells were imaged after 16 hours on a Zeiss ApoTome (Axiovert 40 CFL) using 63 × oil objective. (n = 3); scale: 10 µm. (**C**,**D**) Cytotoxicity of EspF and Map to MDCK cells. Cytotoxicity was assessed by measuring the amount of LDH released by EGFP, EGFP-Tir, EGFP-EspF and EGFP-Map cells into the culture medium. Fold changes in LDH release with respect to EGFP cells are depicted (**C**). Results are representative of three independent experiments. Confluent cultures of untransfected, EGFP, EGFP-Tir, EGFP-EspF and EGFP-Map cells were grown in the absence (0 µM) or presence (50 µM) of cisplatin to induce apoptosis (**D**). The number of viable cells was determined by MTT assay and fold change with respect to untreated cells was plotted for all cell lines. UT: Untransfected.

EspF and Map are targeted to the mitochondria and cause mitochondrial dysfunction^11,27,29^. To exclude the possibility that EspF and Map-associated defects on TJ proteins were due to mitochondria-associated cytotoxity and apoptosis, we checked for the release of cytochrome *c* from the mitochondria in cells expressing EGFP, Tir, EspF and Map (Fig. 7). The cytosolic and mitochondrial fractions of cell lysates were separated by SDS-PAGE and analysed by western blotting. GAPDH and COX IV were used as markers for the cytosolic and mitochondrial fractions respectively. We did not detect cytochrome *c* in the cytosolic fractions indicating that the EGFP-EspF and EGFP-Map proteins did not disrupt the mitochondrial membrane (Fig. 7A). Next, we measured the mitochondrial membrane potential (ΔΨ) in untransfected, EGFP, Tir, EspF and Map cell lines using the JC-1 cationic dye by flow cytometry (Supplementary Figure S2). Since JC-1 dye changes its fluorescence emission from green to orange with increasing ΔΨ, this method provided a useful assay to quantitatively measure the number of cells which displayed shifts in fluorescence emission in these cell lines. We did not find significant differences in the emission shifts in any cell line (Supplementary Figure S2, A–E). One possible reason for EspF and Map cell lines having ΔΨ comparable to untransfected, EGFP and Tir cell lines could be that the genes encoding EspF and Map were tagged at the N-terminus with EGFP which interferes with the mitochondrial localization of these tagged proteins. This was further confirmed by cytotoxicity assays performed to measure viable cells by incubating all cell lines with MTT (Methylthiazolyldiphenyl-tetrazolium bromide). The number of viable cells was counted for each cell line and fold changes were calculated with respect to untransfected cells (normalized to 1). EspF and Map cell lines exhibited similar numbers of viable cells as untransfected, EGFP and Tir cell lines (Supplementary Figure S2F).

We also did not detect any change in the morphology of the mitochondria in cells expressing EspF and Map nor was there any increase in the release of lactate dehydrogenase (LDH) into the medium in cell lines expressing EspF and Map (Fig. 7B,C). Finally, we wanted to exclude the possibility that non-cytotoxic variants of these effectors had been selected during the generation of stable cell lines. We checked all cell lines for sensitivity to cisplatin (*cis*-Diammineplatinum (II) dichloride), a potent cytotoxic drug. We selected cisplatin because MDCK cells are derived from the kidney and cisplatin has been reported to cause nephrotoxicity in cancer patients as well as in cultured LLC-PK1 cells (derived from the proximal tubule of kidney) where it has been reported to induce apoptosis^54^. Confluent cultures of untransfected, EGFP, Tir, EspF and Map cell lines were treated with 50 µM cisplatin for 24 hours and cell viability was measured by MTT assay as described above. The numbers of viable cells were calculated with respect to untreated cells for all cell lines (normalized to 1) and fold change was plotted (Fig. 7D). Although the cell viability was found to decrease in all cell lines, cells expressing EspF or Map exhibited lower viability when treated with 50 µM cisplatin as compared to untransfected, EGFP or Tir cell lines. These data indicated that the expression of EspF and Map increased the sensitivity of these cell lines to cisplatin and also confirmed that these cell lines were not non-cytotoxic variants. These experiments conclusively showed that the depletion of TJ proteins and the concomitant decrease in TER and increase in paracellular flux observed in cells expressing EspF or Map was neither caused by mitochondrial dysfunction nor by loss of cell viability and that both EspF and Map are independently capable of TJ disruption.

## Discussion

EspF and Map have been implicated in causing diarrhea through TJ barrier defects but the precise roles of these effectors on TJ function have not been defined. EPEC infection results in an increase in intestinal permeability and a decrease in the absorption of ions/solutes in the host cells. Earlier studies have shown that multiple functions are disrupted in the host cells by cooperative actions of EPEC effector proteins leading to the rapid onset of diarrhea^55^. For example, EspF cooperates with Map, the bacterial surface protein intimin and its receptor Tir to inactivate SGLT-1^25^ and also inhibits the functions of NHE3^31^. Map inhibits NHERF-1 and cooperates with NleA to inhibit NHERF-2 leading to reduced absorption of sodium ions^32,33^. Additionally, EspG1/G2 dependent down-regulation of the Cl^-^/HCO3^-^ exchanger DRA (down-regulated in adenoma) results in reduced absorption of Cl^-^ in the intestine^56^. TJ disruption is mediated by EPEC effectors EspG1/G2, NleA, EspF and Map. While EspG1/G2 cause a microtubule-dependent disruption of TJs^12^, NleA has been reported to dissociate the TJ proteins occludin and ZO-1 and inhibit transport from the Endoplasmic reticulum to the Golgi thus blocking the delivery of these proteins^13,57^. The effects of Map and EspF on TJs are less clear. As discussed above, some studies have shown that EspF plays a greater role in TJ barrier disruption^26^, other studies have suggested that Map might be more important^11^ while some other studies have shown that cooperative actions of EspF and Map are essential for causing diarrhea^11,25^. Most of these studies were performed using infections of cultured intestinal epithelial cells with the human EPEC strain E2348/69^11,25^ or infections of mice with the mouse strain *C. rodentium*^26^. As EPEC translocates more than 20 effectors into the host cell, one limitation in such models is that the individual functions of a given effector can be masked by the presence of other effectors. For example, in the study conducted by Guttman *et al*.^26^, mutant versions of *C. rodentium* which lacked EspF, did not cause TJ disruption even though Map was present in the infected cells leading the authors to conclude that the junction barrier defects were regulated by EspF and not Map. Other studies, utilizing *in vitro* models of EPEC infection conclusively showed that Map is capable of TJ disruption independently^11^. This was further confirmed in an *in vivo* model utilizing mice infected with *C. rodentium*^24^. Therefore, to examine the individual contributions of EspF and Map in TJ disruption we developed *in vitro* models where these effectors were N-terminally tagged with EGFP and constitutively expressed in MDCK epithelial cells in the absence of all other EPEC effectors. We used cells constitutively expressing EGFP-tagged Tir as controls because Tir has been reported to cooperate with EspF and Map in the onset of diarrhea through the disruption of SGLT-1^25^. We first established the relevance of our EspF and Map *in vitro* models by measuring the permeability of charged and uncharged molecules. Confluent cultures of cell lines expressing EGFP-tagged EspF and EGFP-tagged Map were found to be defective in the passage of both charged ions as well as uncharged tracers. Specifically, the permeability of ions, as measured by TER, was reduced to 0.38 fold and 0.65 fold in EspF and Map cell lines respectively as compared to control cell lines. Further, EspF increased the paracellular flux of small (4 kDa) and large (70 kDa) dextran tracers by ~2.57 fold and ~1.97 fold respectively. Map was found to increase the flux of only the 4 kDa tracer by ~1.55 fold. The constitutive expression of EspF and Map also caused reorganization of the TJ proteins. These data are in agreement with earlier reported studies on EspF and Map conducted using *in vitro* and *in vivo* models^11–13,22–24^ indicating that our *in vitro* MDCK models are comparable to the earlier described models and can be used to understand the process of TJ disruption by EspF and Map in greater detail.

In order to delineate the underlying mechanisms of TJ disruption, we examined *de novo* junction assembly utilizing the calcium switch method where cells are first cultured in low calcium medium to disrupt existing junctions and then switched to normal calcium medium to initiate synchronized junction assembly. The constitutive expression of EspF decreased TER to ~48% and 49% (from 100% in WT cells) in the two EspF cell lines during *de novo* junction assembly and increased the flux of the 4 kDa and 70 kDa dextran tracers by ~3.6 fold and ~2.7 fold respectively. Constitutive expression of Map decreased TER to ~46% and ~48% in the two Map cell lines and the junction permeability increased by ~1.4 fold for 4 kDa dextran and had no effect on the permeability of the 70 kDa tracer. Immunofluorescence studies indicated that EspF and Map expression caused the majority of TJ proteins to accumulate in the cytoplasm. Our data indicates that EspF and Map interfere with junction assembly by preventing the recruitment of these proteins to the junctional complex. Since claudins regulate the passage of charged molecules through the TJ^4,5^, a decrease in TER reflects the loss of claudins at the junctional complex. On the other hand, the passage of non-charged tracers through TJs is regulated by occludin^4,5^. Therefore, it seems likely that while both Map and EspF cause an increase in ion permeability, EspF plays a greater role, as compared to Map, in the increased permeability of non-charged molecules and water during EPEC infections.

We also observed that expression of EspF and Map caused the depletion of TJ proteins as seen by reduced levels of TJ proteins in lysates derived from EspF or Map cell lines. Both effectors were found to deplete claudin-1, claudin-4 and occludin but the depletion of ZO-1 was not observed either in EspF or Map cell lines. In order to establish whether this depletion occurred at the TJ complex, we labeled the cell surface proteins with biotin and separated the labeled membrane proteins by binding with immobilized streptavidin resin. These experiments revealed that claudin-1, -4, occludin as well as ZO-1 was depleted in the membrane fractions. It is unlikely that the depletion of TJ proteins by EspF and Map reflects a general down-regulation of proteins in host cells as neither β-actin nor E-cadherin (a protein associated with adherens junctions) was found to be down-regulated in our study.

At the transcription level, both effectors decreased the expression of *claudin-1* although this effect was more pronounced for EspF. Interestingly, Map did not inhibit the transcription of *claudin-4*, *occludin* and *ZO-1*. On the other hand, EspF significantly interfered with the transcription of *occludin* and *ZO-1* and to a lesser extent of *claudin-4*. To analyze the fate of the existing TJ proteins in EspF and Map cell lines, we first blocked protein synthesis by cycloheximide treatment and then assessed the total levels of these proteins. EspF and Map were found to affect protein stability as seen by a 50% decline in the total levels of the TJ proteins within 3 to 6 hours of cycloheximide treatment as opposed to 9 to 12 hours in control cell lines. Additionally, inhibition of lysosomal degradation was found to increase the levels of claudin-4 and occludin in the Map cell lines and to a lesser extent in the EspF cell lines. Lysosomal inhibition had no effect on the levels of claudin-1 in either the EspF cell lines or the Map cell lines suggesting the claudin-1 was regulated at the transcriptional level by both EspF and Map. This data indicated that EspF-mediated depletion of claudin-4 and occludin was regulated at both the transcription and translation level while Map expression caused the depletion of claudin-4 and occludin by lysosomal degradation. To our knowledge, this is the first report of the involvement of Map in the lysosomal degradation of TJ proteins. A recent study has revealed that EspF causes the depletion of DNA mismatch repair proteins through post-transcriptional mechanisms implicating EspF in the promotion of colorectal carcinogenesis^58^. Our data provides additional support for a role of EspF in modulating gene expression.

In co-immunoprecipitation assays, we identified that EspF interacted with ZO-1 and actin. It has been reported that EspF, derived from rabbit EPEC, causes the accumulation of TJ proteins ZO-1 and ZO-2 in actin pedestals in infected cells and was also found in the co-immunoprecipitation complex with these proteins during the infection of cells with rabbit EPEC^42^. More recently, a study on the Enterohemorrhagic E. coli (EHEC) - host interactome, identified ZO-1 as a binding partner for EHEC-derived EspF in yeast two hybrid screens^59^. Our findings confirm this interaction as we also found that EspF from the human EPEC strain E2348/69 interacts with ZO-1. Whether this interaction is direct or indirect remains to be determined and requires further work. ZO-1 contains an actin-binding domain within its C-terminus and its interaction with actin has been reported to play an important role in stabilizing the TJ solute barrier^8^. We speculate that EspF may interfere with the interaction between ZO-1 and actin to destabilize the TJ barrier. Whether EspF, ZO-1 and actin are part of the same complex or whether EspF forms separate complexes with ZO-1 and actin and the functional consequence of these interactions requires further investigation. Additionally, we report, for the first time, that Map interacts with non-muscle myosin II heavy chain isoforms A, B and C. Map was also found to form a complex with actin which is consistent with data reported in earlier studies^43^. TJ assembly is regulated by the differential modulation of the actin cytoskeleton and the motor protein myosin^53^. Cytoskeletal reorganization and acto-myosin contractility directly affects the junctional barrier through myosin II activation and several TJ proteins have been reported to regulate this signaling pathway through the modulation of Rho GTPases^4,7^. Interestingly, Map has been reported to activate Cdc42, a member of the Rho GTPase family, by acting as an exchange factor for Cdc42 leading to the formation of transient filopodia^28,60^. The C-terminus of Map interacts with the PDZ domain of Ebp50 (ezrin binding protein 50) and this interaction subsequently causes the activation of Cdc42 at the bacterial docking site^43^. The Map-Ebp50 interaction acts as a scaffold linking Map with actin and causing localised actin polymerization^43^. Cdcc42 has been reported to regulate TJ assembly and the transport of membrane proteins in MDCK cells^61^. Earlier studies have revealed that EPEC induced phosphorylation of the 20-kilodalton myosin light chain (MLC20) is responsible for the perturbation of the intestinal barrier^62^. However, the effector protein(s) responsible were not identified in this study. Since, the phosphorylation of MLC and the subsequent actinomyosin contractility regulates the TJ barrier^4,7,53^, it is tempting to speculate that Map modulates this pathway to disrupt TJs. Whether the interaction of Map with non-muscle myosin II isoforms is linked to the complex it forms with ebp50 and whether Map targets this pathway to disrupt TJs through the regulation of Cdc42 remains to be investigated.

## Conclusions

Frequent bouts of diarrhea caused by EPEC infections are associated with severe morbidity and mortality in young children especially in developing countries. EPEC infection causes an acute loss of water and electrolytes in patients due to increased permeability through TJs leading to dehydration and death. Currently, the therapies used for treatment rely on the administration of antibiotics to eliminate the bacteria and oral rehydration solutions to restore the electrolyte balance. However, these therapies are ineffective in blocking the leakage through TJs. In this report, we have used a simple bacteria-free model to demonstrate that the EPEC effectors EspF and Map can independently disrupt TJs. This model can serve as a tool for designing effective strategies to close the TJ barrier in EPEC infections. Using this model, we report here that EspF and Map use a multi-pronged strategy to disrupt TJs during EPEC infections by (i) preventing the junctional recruitment of TJ proteins by accumulating them in the cytoplasm, (ii) depleting the junctional proteins through transcriptional and post-transcriptional mechanisms and (iii) interacting with ZO-1 (EspF) and myosin II (Map) to regulate the assembly and disassembly of tight junctions ultimately compromising barrier integrity. Our data also conclusively shows that TJ barrier disruption by EspF and Map does not require the actions of the bacterial surface protein Intimin or its receptor Tir nor is it linked to mitochondrial dysfunction caused by EspF and Map. In conclusion, our data indicates that independent actions of EspF and Map are capable of promoting permeability through TJs. Further, the interactions between EspF-ZO-1 and Map-myosin II as well as the differential regulation of transcriptional and post-transcriptional mechanisms suggest that these effectors have non-overlapping functions and may target distinct signaling pathways to cause maximum disruption to the TJ barrier. A recently published study, which revealed that EspF, but not Map, promotes the endocytosis of Crb3 (Crumbs) disrupting cell polarity^40^, provides further support to our hypothesis that Map and EspF have distinct functions in infected epithelial cells.

## Methods

### Plasmids

The genes encoding EspF and Tir were amplified from the genomic DNA of EPEC O127:H6 strain E2348/69 by PCR using the following primers:

- EspF Forward: 5′-AAAAATCTAGAGTCGACCCCTTTCTTCGATTGCTCATAGG-3′
- EspF Reverse: 5′-AAAAAGGATCCCTTAAGATGGTTAATGGAATTAGTAACGCTG-3′
- Tir Forward: 5′-AAAAATCTAGAGTCGACAACGAAACGTACTGGTCCCGG-3′
- Tir Reverse: 5′-AAAAAGGATCCCTTAAGATGGCTATTGGTAACCTTGGT-3′

The resulting PCR products were digested with BamHI and SalI and inserted into the compatible ends of the pEGFP-C1 vector between the BglII and SalI sites to generate an N-terminal EGFP-tagged EspF or N-terminal EGFP-tagged Tir construct for expression in MDCK cells. The same PCR product was inserted into the pGEX-4T-3 vector between the BamHI and SalI sites to generate N-terminal GST-tagged EspF and Tir constructs for use in pull-down assays. The EGFP- and GST-tagged Map constructs were made as described earlier^41^.

### Cell culture

MDCK II cells were cultured in DMEM (Dulbecco’s Modified Eagle Medium) supplemented with 10% FBS (fetal bovine serum) and 1X penicillin-streptomycin-amphotericin B solution (HiMedia Laboratories). T84 cells were cultured in DMEM/F-12 (Dulbecco’s Modified Eagle Medium/Nutrient Mixture F-12) supplemented with 10% FBS and 1 × penicillin-streptomycin-amphotericin B solution. MDCK cell lines with stable integration of pEGFP-*EspF*, pEGFP-*Tir* and pEGFP-C1 vector alone were generated by us using the calcium phosphate protocol described earlier for the generation of stable cell lines of pEGFP-*Map*^41^. Cell lines expressing EGFP, Tir, EspF and Map were selected and maintained in medium containing G418 (used at a final concentration of 500 µg/ml). All cell lines were maintained in a humidified CO_2_ incubator at 5% CO_2_ at 37 °C. Two independent clones were used for each cell line in all experiments.

### Transepithelial resistance and dextran permeability

The cells were trypsinized and 1 × 10^5^ cells were seeded on 12-mm polycarbonate Transwell filters with a pore size of 0.4 µm (Corning) in DMEM containing 10% FBS, 1X penicillin-streptomycin-amphotericin B solution and G418 (for EGFP, Tir, EspF, and Map cell lines) and allowed to grow until confluent with medium changes every alternate day. Transepithelial resistance was measured daily using a voltohmmeter (Millicell ERS; EMD Millipore Corporation). All cell lines attained maximum TER values on the 7th day of plating on filters. To obtain the final TER values (Ωxcm^2^), the resistance of the blank filter was subtracted from the resistance of the filters containing cell lines and multiplied with the surface area of the 12-mm filters (1.12 cm^2^). Once the cultures displayed maximum TER values, the permeability of uncharged tracers across cell monolayers was measured by adding 1 mg/ml of 4 kDa FITC-Dextran and 70 kDa Rhodamine B-Dextran (Sigma-Aldrich) to the upper chamber. After 5 hours incubation at 37 °C, aliquots of 100 µl were removed from the lower chamber and fluorescence was measured using a Fluorometer (λ excitation 490 nm and λ emission 520 nm for 4 kDa FITC-Dextran; λ excitation 570 nm and λ emission 595 nm for 70 kDa Rhodamine B-Dextran). Each 12 well plate (containing Transwell filters) included untransfected cells as controls and fold changes in TER or flux for EspF and Map cell lines were calculated with respect to untransfected cells grown on the same plate(s).

### Calcium switch Assays

For *de novo* junction assembly, confluent cultures of untransfected, EGFP, EGFP-Tir, EGFP-EspF or EGFP-Map cells were trypsinized and resuspended in low calcium (<5 µM) medium (S-MEM supplemented with 10% dialyzed FBS, 1 mM sodium pyruvate, 2mM L-glutamine and 1% Penicillin-Streptomycin (for untransfected cells) and 500 μg/ml G418 for (EGFP, EGFP-Tir, EGFP-EspF and EGFP-Map cells) and plated at a density of 2 × 10^6^ cells**/**ml on permeable 12-mm Transwell filters with a pore size of 0.4 μm (Corning). After 18–20 hours, the cells were incubated in normal calcium (1.8 mM) medium. TER measurements were started 30 minutes later and then recorded hourly until junction assembly was complete using a voltohmmeter (Millicell ERS; EMD Millipore Corporation). TER was calculated by subtracting the resistance of the blank filters from the resistance values of filters containing cell lines. After 24 hours, the permeability of 4 kDa FITC-Dextran and 70 kDa Rhodamine B-Dextran (Sigma-Aldrich) was measured as described above. Each 12 well plate (containing Transwell filters) included untransfected cells as controls and TER or flux values were calculated with respect to untransfected cells grown on the same plate(s).

### Immunofluorescence assays

Untransfected, EGFP, EGFP-Tir, EGFP-EspF and EGFP-Map cells were cultured on glass cover-slips until confluent and fixed with chilled methanol for 5 minutes at −20 °C, rehydrated with PBS for 5 minutes at room temperature and blocked with PBS containing 0.5% BSA at room temperature for 30 minutes. Cover slips were incubated with primary antibodies against TJ proteins for 2 to 4 hours. Primary antibodies, used at 1:300 dilution, were: claudin-1 (Thermo Fisher Scientific, #374900, mouse monoclonal), claudin-4 (Thermo Fisher Scientific, #329400, mouse monoclonal), occludin (Thermo Fisher Scientific, #331500, mouse monoclonal) and ZO-1 (Thermo Fisher Scientific, #339100, mouse monoclonal). After washing three times with blocking solution, the cover slips were incubated with Cy3-conjugated secondary antibody (Millipore) for 1 hour at room temperature. Cover-slips were mounted in ProLong Diamond Antifade mountant (Thermo Fisher Scientific, #P36961). Images were acquired at 63X magnification on a Zeiss ApoTome (Axiovert 40 CFL).

### Preparation of total protein lysates

Total protein lysates were prepared from confluent cultures of untransfected, EGFP, EGFP-Tir, EGFP-EspF and EGFP-Map cells grown either on 6-well plates or on Transwell filters (Corning). Cell lysates were prepared by adding 1X Laemelli buffer to the cells and passing the mixture through a 23-gauge needle several times. The lysates were electrophoresed on 12% SDS polyacrylamide gels. Western blotting was performed using above listed monoclonal antibodies against TJ proteins at 1:1000 dilution. The results obtained after probing with monoclonal antibodies were also confirmed by using polyclonal antibodies. The antibodies used were claudin-1 (Thermo Fisher Scientific, #717800, rabbit polyclonal), Occludin (Thermo Fisher Scientific, #711500, rabbit polyclonal), ZO-1 (Thermo Fisher Scientific, #617300, rabbit polyclonal), pan-cadherin (Thermo Fisher Scientific, #717100, rabbit polyclonal), actin (Cell Signaling Technology, #3700S, mouse monoclonal; and #A2066, rabbit polyclonal, Sigma-Aldrich) and GFP (#2955, mouse monoclonal, Cell Signaling Technology and #G1544, rabbit polyclonal, Sigma-Aldrich). Rabbit anti-GAPDH antibody (1:10,000 dilution; BioBharti, #BB-AB0060) was used as a loading control to ensure equal loading of cell lysates on gels. The x-ray films were scanned and band intensities were quantitated using ImageJ software. For the inhibition of protein synthesis, confluent cells, cultured in 6 well plates, were incubated in the absence (−) or presence (+) of cycloheximide (Sigma-Aldrich) at a concentration of 25 μg/ml for 3, 6, 9 and 12 hours prior to cell lysis. Subsequently, the cell lysates were made by adding 1X Laemelli buffer and analyzed by SDS-PAGE as described above. For the inhibition of lysosomal and proteasomal degradation, confluent cells were incubated in the absence or presence of 100 µM chloroquine or 10 µM MG132 respectively for 18 hours. Cell lysates were made by adding 1X Laemelli buffer and analyzed by SDS-PAGE.

### Biotinylation Assays

The HOOK Cell Surface Protein Isolation kit (G‐Biosciences) was used to label cell surface proteins with Sulfo‐NHS‐SS‐Biotin, a water‐soluble biotinylation reagent that has an *N*‐hydroxysulfosuccinimide (sulfo‐NHS) ester. Briefly, untransfected, EGFP, Tir, EspF and Map cell lines were cultured in 100-mm plates until 90–95% confluent, washed twice with ice cold PBS and treated with 3 ml per plate of biotin solution (2 mg/ml biotin reagent in PBS) for 30 minutes at 4 °C with shaking. The cells were washed twice with cold 100 mM Glycine (in PBS) for 5 minutes with gentle rocking, twice with cold 20 mM Glycine (in PBS) for 5 minutes and twice in cold PBS for 5 minutes. The cells were then lysed in 200 μl of lysis buffer (containing 50 mM Tris/HCl (pH 7.4), 150 mM NaCl, 1 mM EDTA, 1% (w/v) Triton X-100 and 1X protease inhibitor cocktail). The cell lysates were passed through a 22-gauge needle five times and rotated at 4 °C for 30 minutes followed by centrifugation at 12,000 × g for 15 minutes. After removing 100 µl as input, the supernatant was incubated with 50 µL of streptavidin-agarose beads overnight at 4 °C. The samples were then centrifuged at 12,000 × g for 30 seconds at 4 °C and the supernatant was discarded. The beads were washed once with lysis buffer, twice with buffer containing 0.1% Triton X-100 (in PBS, pH 7.4), 350 mM NaCl, 5 mM EDTA and twice with PBS. Samples were analysed by SDS-PAGE and western blotting using the antibodies described above. Fold changes were calculated by densitometric scanning of films using ImageJ software.

### Quantitative real time PCR (qRT-PCR)

Cells expressing EGFP, EGFP-Tir, EGFP-EspF and EGFP-Map were grown in 100-mm plates until confluent. Total RNA was isolated using the PureLink RNA Mini Kit (Ambion). cDNA synthesis and qRT-PCR assays were performed using the TaqMan Gene Expression Cells-to-C_T_ Kit (Applied Biosystems) according to the manufacturer’s protocol. The RT-PCR reaction was carried out at 37 °C for 60 minutes and 95 °C for 5 minutes in a 50 μL volume using 1 µg of total RNA. Negative controls without the 20X RT Enzyme mix were kept for all samples. For qPCR, 2 μL of cDNA from each sample was used in a 10 μL reaction volume containing 5 μL of 2X TaqMan Gene Expression Master Mix and 0.5 μL of 20X TaqMan Gene expression assay. The cycling conditions used were: 50 °C for 2 minutes; 95 °C for 10 minutes; and 40 cycles of 95 °C for 15 seconds and 60 °C for 1 minute. All reactions were set up in triplicate for the target genes and the endogenous control gene. TaqMan gene expressions assays for *claudin-1, claudin-4*, *occludin*, *ZO-1*, and the endogenous control gene *GAPDH* were purchased from Life Technologies Ltd. (Assay Id: Cf02713195_u1, Cf02695489_s1, Cf02624085_m1, Cf02628478_m1 and Cf04419463_gH respectively). The test and the calibrator samples were normalized with their endogenous controls and relative quantitation of gene expression was determined by calculating the fold difference between EGFP cells and EGFP-Tir, EGFP-EspF or EGFP-Map cells by applying the ΔΔC_T_ method^51^.

### Co-Immunoprecipitation assays

Confluent monolayers of untransfected, EGFP-EspF or EGFP-Map cells, grown in a 100-mm plate, were washed with cold PBS and suspended in RIPA buffer (1% Empigen BB, 1% triton X-100, 0.5% sodium deoxycholate, 0.2% SDS, 150 mM NaCl, 20 mM HEPES, 2 mM EDTA, 0.2 mM PMSF and 1 × protease inhibitor cocktail). After extraction by passing through a 21-gauge needle several times, the mixture was clarified by centrifugation and the supernatant was incubated with 2 µg of GFP antibody (#G1544, Sigma-Aldrich) overnight at 4 °C to precipitate the protein-antibody complex. Approximately 50 µl of Protein G-Agarose beads (Sigma-Aldrich) were added to the lysates and mixed for 4 hrs at 4 °C for immobilization of protein-antibody complex on the beads. The beads were centrifuged at 10,000 × g for 30 seconds and the supernatant was discarded. The beads were washed twice with RIPA buffer and once with PBS, boiled for 5 minutes and subjected to SDS-PAGE and western blot analysis. Blots were probed with antibodies against Myosin IIA (#3403, rabbit polyclonal, Cell Signaling Technology), Myosin IIB (#3404, rabbit polyclonal, Cell Signaling Technology), Myosin IIC (#8189, rabbit monoclonal, Cell Signaling Technology), GFP (#2955, mouse monoclonal, Cell Signaling Technology), actin (#3700, mouse monoclonal, Cell Signaling Technology) and ZO-1 (#339100, mouse monoclonal, Thermo Fisher Scientific) at 1:1000 dilution.

### Mitochondrial Assays

The mitochondrial and cytosolic fractions of cells were obtained by incubating confluent cells derived from untransfected, EGFP, Tir, EspF and Map cell lines in buffer containing 200 µg/ml digitonin, 80 mM KCl (in PBS) on ice for 5 minutes. The cell lysates were centrifuged at 800 × g for 5 minutes and the supernatant (cytosolic fraction) was collected. The pellet was resuspended in lysis buffer (50 mM Tris-HCl, pH 7.4, 150 mM NaCl, 2 mM EGTA, 2 mM Na_2_EDTA, 0.2% (w/v) Triton X-100, 0.2% IGEPAL CA-630 and 1 × protease inhibitors) and incubated for 10 minutes at 4 °C. The solution was centrifuged at 10,000 × g for 10 minutes to collect the mitochondrial fraction. Equal amounts of cytosolic and mitochondrial fractions were subjected to SDS-PAGE and analyzed by western blotting with antibodies against cytochrome *c* (#11940, rabbit monoclonal, Cell Signaling Technology), Cox IV (#11967, mouse monoclonal, Cell Signaling Technology) and GAPDH (#BB-AB0060, rabbit polyclonal, BioBharti). Cox IV and GAPDH were used as loading controls for the mitochondrial and cytosolic fractions respectively. Mitochondrial membrane potential was analyzed by using the JC-1 dye (5,5′,6,6′-Tetrachloro-1,1′,3,3′-tetraethylbenzimidazolocarbocyanine iodide; Sigma-Aldrich; #T4069) following the manufacturer’s protocol. Briefly, ≥ 2 × 10^5^ cells were harvested and the total volume was adjusted to 1 ml with pre-warmed (37 °C) complete cell culture medium. JC-1 was added at a final concentration of 2.5 μg/ml while vortexing until the dye was dissolved and a uniform red-violet colour was obtained. Subsequently, the samples were incubated at 37 °C for 10 minutes in the dark. The cells were then washed twice with PBS and centrifuged for 5 minutes at 500 × g at room temperature and analysed by flow cytometry. A minimum of 10,000 cells for each sample were analyzed by BD FACSCalibur flow cytometer. The emission wavelengths of JC-1 monomers and ‘J-aggregates’ were 525 nm (Fl1 channel) and 585 nm (Fl2 channel).

### Cytotoxicity Assays

Untransfected, EGFP, EGFP-Tir, EGFP-EspF and EGFP-Map cells were grown in 96 well plates (approximately 1 × 10^4^ cells per well) in triplicate until confluent. MTT (Methylthiazolyldiphenyl-tetrazolium bromide, #M2128, Sigma-Aldrich) was added at a final concentration of 0.5 mg/ml and the plate was incubated in the dark for 3 hours. Subsequently, the cells were washed with PBS, the residue was dissolved in DMSO for 5 minutes and absorbance was measured at 595 nm. For LDH measurements, we used the CytoScan‐Fluoro Assay kit (G-Biosciences). Approximately, 1 × 10^4^ cells from each cell line were cultured in triplicate wells of a 96-well plate for 24 hours. The plates were removed from the 37 °C incubator and equilibrated to 22 °C for about 20‐30 minutes following which 100 µl of CytoScan‐Fluoro Reaction Buffer was added to 100 µl medium containing cells and incubated at 22 °C for 10 minutes. The reaction was stopped by adding 50 μl of CytoScan‐Fluoro‐Stop Solution to each well and the plate was shaken for 10‐15 seconds. Fluorescence was measured with an excitation wavelength of 560 nm and an emission wavelength of 590 nm. For positive controls, cells grown in triplicate wells were lysed in 2 μl of CytoScan‐Fluoro‐Lysis Buffer per 100 μl original volume to generate maximum LDH Release Controls. The fluorescence of the culture medium was subtracted from the values obtained in experimental samples and positive controls and expressed as fold change with respect to the values obtained for EGFP cell lines (normalized to 1). Cell viability was determined similarly by culturing all cell lines (approximately 1 × 10^4^ cells) in triplicate wells of a 96 well plate in the absence or presence of cisplatin (#P4394, Sigma-Aldrich) at a concentration of 50 µM for 24 hours. The number of viable cells, after cisplatin treatment, was estimated by MTT assay as described above.

### Transient Transfection and Transduction

T84 cells were grown on glass cover-slips in 24 well plates until 80% confluent and transfected with pEGFP-C1 vector, pEGFP-Tir, pEGFP-EspF and pEGFP-Map plasmids using Lipofectamine 2000 reagent (Thermo Fisher Scientific, # 11668027) according to the manufacturer’s protocol. Briefly, 500 ng of each plasmid construct was mixed with Lipofectamine 2000 reagent in 1:1 ratio and incubated for 20 minutes at room temperature. Subsequently, the mixture was added to the cells and incubated overnight in a CO_2_ incubator. The cells were processed for immunofluorescence and probed with antibodies against TJ proteins as described above. For examining the morphology of the mitochondria, we used the CellLight Mitochondria-RFP, BacMam 2.0 construct (#C10601, Thermo Fisher Scientific) to label mitochondria with red fluorescent protein (RFP) in live cells. This fusion construct contains the leader sequence of E1 alpha pyruvate dehydrogenase and RFP-tag packaged in the baculovirus. Cells from untransfected, EGFP, Tir, EspF and Map cell lines were transduced with 2 μL of BacMam 2.0 reagent (per 10,000 cells in media), incubated for 16 hours at 37 °C and images were acquired at 63 × magnification on a Zeiss ApoTome (Axiovert 40 CFL).

### Statistical Analysis

All experiments were performed at least three times with two independent clones (biological replicates) for EGFP, EGFP-Tir, EGFP-EspF and EGFP-Map. Comparisons between groups were made using one-way analysis of variance (ANOVA) and post-hoc Holm-Bonferroni corrected t-tests. Differences between groups were considered significant at p-values < 0.05.

## Electronic supplementary material

Supplementary File
