# Supplementary material for: Enteropathogenic E. coli effectors EspF and Map independently disrupt tight junctions through distinct mechanisms involving transcriptional and post-transcriptional regulation
Source: Sci Rep. 2018 Feb 27;8:3719. doi: 10.1038/s41598-018-22017-1 (PMC5829253; doi:10.1038/s41598-018-22017-1)
Supplement: Supplementary file 1 — Supplementary File [file 41598_2018_22017_MOESM1_ESM.doc]

**Supplementary Information**

**Enteropathogenic *E. coli* effectors EspF and Map independently disrupt tight junctions through distinct mechanisms involving transcriptional and post-transcriptional regulation.**

Anand Prakash Singh^#^, Swati Sharma^#^, Kirti Pagarware^#^, Rafay Anwar Siraji^#^, Imran Ansari^#^, Anupam Mandal, Pangertoshi Walling and Saima Aijaz*****

^#^Equal Contribution

*****Corresponding Author

**Affiliations:**

Centre for Molecular Medicine,

Jawaharlal Nehru University,

New Delhi 110067

India

**Address of Corresponding Author:**

Saima Aijaz

Centre for Molecular Medicine,

Jawaharlal Nehru University,

New Delhi 110067

India

Telephone: +91-11-26738733

Email: [s_aijaz@mail.jnu.ac.in](mailto:s_aijaz@mail.jnu.ac.in)

**Supplementary Figure S1**

**Supplementary Figure S2**

**Supplementary Figure S3**

**Supplementary Figure S4**

**Supplementary Figure S5**

**Supplementary Figure S6**

**Supplementary Figure S7**

**Supplementary Figure S8**

**Supplementary Figure Legends**

**Supplementary Figure S1. Transient transfection of T84 cells with plasmid constructs expressing EGFP, Tir, EspF and Map.** T84 cells, grown to 80% confluency, were transiently transfected with plasmid constructs for the expression of EGFP, EGFP-Tir, EGFP-EspF and EGFP-Map. The cells were fixed and stained for primary antibodies against claudin-1, claudin-4, occludin and ZO-1 for 2 hours followed by labeling with Cy3-conjugated secondary antibody. Nucleus was stained with DAPI; scale: 10 µm.

**Supplementary Figure S2. Measurement of mitochondrial potential and cytotoxicity.** (A-E) Untransfected cells or cells expressing EGFP, EGFP-Tir, EGFP-EspF and EGFP-Map were treated with JC-1 and analyzed by flow cytometry to measure mitochondrial membrane potential. The fluorescence pattern of the indicated cell lines is shown. Untransfected cells and cell lines expressing EGFP alone were used as negative controls and for gating correction. (F) The indicated cell lines were treated with MTT to measure cell viability. Shown are the fold changes with respect to untransfected cells (normalized to 1). Data represents means ± s.e.m; UT: Untransfected.

**Supplementary Figure S3. Uncropped western blots showing the depletion of tight junction proteins in cell lines constitutively expressing EspF and Map.** Cell lysates derived from untransfected (UT), EGFP, EGFP-Tir, EGFP-EspF and EGFP-Map cell lines were separated by electrophoresis on 12% SDS-polyacrylamide gels and transferred to PVDF membranes. Equal amounts of cell lysates were loaded after normalizing with GAPDH. Blots were probed with the indicated primary antibodies and HRP-conjugated secondary antibodies. Samples were obtained from the same experiment and all gels were processed in parallel.

**Supplementary Figure S4. Uncropped western blots showing biotin-labeled cell surface tight junction proteins.** The cell surface proteins were labeled with membrane impermeable Sulfo-NHS-Biotin and immobilized on streptavidin-agarose beads. The beads were analyzed by SDS-PAGE. E-cadherin was used as a loading control for cell surface proteins. Samples were obtained from the same experiment and all gels were processed in parallel.

**Supplementary Figure S5. Uncropped western blots showing the levels of tight junction proteins after cycloheximide treatment.** Confluent cultures of untransfected cells or cells expressing EGFP-Tir, EGFP-EspF and EGFP-Map were treated with cycloheximide (25μg/ml) for 3, 6, 9 and 12 hours prior to cell lysis and analyzed by immuno-blotting with the indicated primary antibodies. Samples were obtained from the same experiment and all gels were processed in parallel.

**Supplementary Figure S6. Uncropped western blots showing the effect of the lysosomal inhibitor chloroquine on the amounts of tight junction proteins.** Confluent cultures of cells were treated with 100µM chloroquine for 18 hours and cell lysates were analyzed by immuno-blotting with the indicated antibodies. Experiments were performed three times and representative blots are shown. UT: Untransfected.

**Supplementary Figure S7. Uncropped western blots showing the interaction of EspF and Map with distinct tight junction regulatory proteins.** Confluent cultures of untransfected, EGFP-EspF or EGFP-Map cells were lysed in 1X RIPA buffer and the supernatant was incubated with rabbit anti-GFP antibody overnight at 4°C. Interacting proteins were immuno-precipitated by mixing with 50 µl of protein G-agarose beads for 4 hours at 4°C. After washing, the beads were subjected to western blot analyses (n=3). Blots were probed with indicated primary antibodies. Experiments were performed three times and representative blots are shown. UT: Untransfected.

**Supplementary Figure S8. Uncropped western blots showing that tight junction disruption by EspF and Map is independent of mitochondrial function.** Cytosolic and mitochondrial fractions were isolated from untransfected, EGFP, EGFP-Tir, EGFP-EspF and EGFP-Map cell lines and analyzed by immuno-blotting with cytochrome *c* antibody. Equal loading on gels was confirmed by using COX IV as the mitochondrial marker and GAPDH as the cytosolic marker. Blots are representative of three independent experiments.
